# A systematic review and meta-analysis on the prevalence of extended-spectrum beta-Lactamase-Producing *Klebsiella Pneumonia* in Nigeria

**DOI:** 10.4314/ahs.v24i3.5

**Published:** 2024-09

**Authors:** Christian Kelechi Ezeh, Daniel Chinonso Digwo, Irene Amara Okeke, Prisca Chidinma Elebe, Emmanuel Onyedikachi Ezeh

**Affiliations:** 1 Department of Microbiology, University of Nigeria, Nsukka, Enugu State, Nigeria; 2 Department of Biochemistry, University of Nigeria, Nsukka, Enugu State, Nigeria

**Keywords:** Extended spectrum beta-latamase, *Klebsiella* Pneumonia, meta-analysis, Nigeria

## Abstract

**Background:**

Rapid emergence and proliferation of extended spectrum beta-lactamase producing *Klebsiella* pneumoniae (ESBL-KP) constitute a serious health problem globally.

**Objectives:**

The aim of this study was to evaluate the pooled prevalence of ESBL-KP and ESBL genes in Nigeria.

**Methods:**

A quantitative method (Meta-analysis) design was used to summarize pooled results of primary studies. Different databases [Google Scholar, PubMed, and African Journal Online (AJOL)] were searched for relevant studies. Meta-analysis was done using random-effects model. I2 and Egger test was used to ascertain heterogeneity and publication bias evaluation.

**Results:**

Eighteen observational studies were selected and the pooled prevalence of ESBL-KP in Nigeria was 47.3% [95% confidence interval (CI) 37 – 58%]. Among the genes encoding ESBL, OXA had the highest pooled prevalence in the selected studies [57% (95% CI 32, 76)], followed by TEM [55% (95% CI 36, 70)], CTX-M [54% (95% CI 38, 70)], and 41% (95% CI 27, 57). Heterogeneity tests (I2) was observed to be between 69.22 and 95.63 % for ESBL-KP and ESBL genes in the studies. Egger tests showed no publication bias (0.09 – 0.99).

**Conclusions:**

This meta-analysis demonstrated that the prevalence of ESBL-KP is increasing in Nigeria. Hence, antimicrobial stewardship and infection control measures for the prevention and spread of these strains be implemented.

## Introduction

*Klebsiella pneumoniae* is a Gram-negative, facultative, non-motile, lactose fermenting and encapsulated rod shaped bacterium associated with infections such as pneumonia, urinary tract infection, meningitis, skin and soft tissue infection, bloodstream infection, pyogenic liver abscess and intra-abdominal infection in both hospital and community settings 1, 2. The rapid emergence and proliferation of antimicrobial resistance in *K. pneumoniae* has become a significant public health concern 3. Indiscriminate use of antibiotics both in the community and hospital settings have resulted to increase in selective pressure on *K. pneumonia*, leading to the proliferation of antibiotic resistance^4^. Various antibiotics have been used to treat infections caused by *Klebsiella pneumoniae* of which the beta-lactam antibiotics constitutes the bulk 3.

Beta-lactams antibiotics are one of the most routinely used drugs; these antibiotics can induce the production of beta-lactamases in *K. pneumonia* 5. Among beta-lactamases, extended-spectrum beta-lactamses (ESBLs) are one of the most widely spread resistance mechanisms. These enzymes confer resistance to *K. pneumonia* against Beta-lactams class of antibiotics (monobactams, penicillins, and cephalosporins) by hydrolyzing the bet-lactam rings 6, 7. Most of these enzymes were formed through spontaneous mutation of reduced spectrum β-lactamases^8^. Furthermore, ESBLs are mostly encoded by popular genes such as blaTEM, blaSHV and blaCTX-M^9^. These genes just like other types of genes are usually found in the mobile genetic elements and are transferred among bacterial species 10. OXA (oxacillinase), PER (Pseudomonas extended-resistant), (Guyana extended-spectrum-lactamase) and VEB (Vietnamese extended-spectrum – lactamase are ESBLs that are less studied.

Klebsiella pneumonia producing ESBL (ESBL-KP) was first reported in Europe and USA in 1983 and 1989 respectively 6. There is widespread dissemination of ESBL producing bacteria worldwide. These organisms are more severe in developing nations. Infections caused by ESBL-KP pose a serious health concern because of the limited attention paid to them especially in developing countries like Nigeria. Also, ESBL-KP strains are found to be multidrug resistant due to their ability to resist other classes of antibiotics including aminoglycosides, trimethoprim/sulfamethoxazole, and fluoroquinolones 7.

Many studies in Nigeria also detected ESBL-KP genes; however, the dissemination of these genes in Nigeria is not well known due to ineffective detection methods and negligence of ESBL-KP as a public health problem. Various studies on the prevalence of ESBL-KP have been reported in various parts of Nigeria. The current review add to the systematic review by Nuhu et al.^11^, who focused on the prevalence of ESBL among the Enterobacteriaceae family. Hence, this review focused on determining the pooled prevalence of ESBL-KP and genes in Nigeria.

## Methods

### Study design and literature search

A quantitative method (Meta-analysis) design was used to summarize pooled results of primary studies on prevalence of ESBL-KP and genes in Nigeria. The study is a country wide study in which primary research on the prevalence of ESBL-KP and genes from all the geo-political zones of Nigeria are included. Electronic databases including Google scholar, PubMed and African Journal OnLine (AJOL) were used for literature search from 2000 to July 2022 using the combinations of the following keywords: “Klebsiella pneumonia”, “K. pneumonia”, “extended-spectrum β-lactamases”, ESBLs”, and “Nigeria”. Boolean operators “OR” and “AND” were used for keywords combination were appropriate. In addition, manual search for relevant articles from references of already published work were conducted.

### Eligibility criteria

Inclusion criteria for articles retrieved from literature search which were considered eligible for inclusion include: studies that uses phenotypic detection of ESBL production such as double disk synergy test (DDST), E test, disc replacement method (DRM) based on Clinical and Laboratory Standards Institute (CLSI) guidelines 12, studies that used molecular methods for detection of ESBLs genes, studies depicting the prevalence of ESBL-KP, studies that used human samples, and studies written in English language. Studies that were not considered eligible include: studies that did not report the prevalence of ESBL-KP or ESBLS gene, studies on non-human samples, studies not in Nigeria, and studies not written in English language. To ensure precise reporting of relevant data, the Preferred Reporting Items for Systematic Review and Meta-analysis (PRISMA) guidelines was used.^13^,^14^.

### Study selection and data extraction

Review of titles and abstracts of articles were independently done by two reviewers (CKE and DCD) for possible inclusion based on the eligibility criteria. Furthermore, full text articles were also screened for further confirmation. Where there are varying opinions, both reviewers settle it through discussion. Relevant data extracted from the included studies include: leading author's name and year of publication, study place, study period, isolate source, prevalence of ESBL-KP, ESBL encoding genes, and method of ESBL detection. A table was created to input the data for clarity. [U1]Quality assessment of included studies was done based on Joanna Briggs institute (JIB) checklist 10. Two reviewers (CKE and CPE) answered ten questions in which a “YES” is given as one point and any disagreement was resolved through discussion. A 7-10 score range was used as a pass score for included articles.

### Statistical analysis

Comprehensive Meta-analysis Software V3 was used for meta-analyses. Due to the heterogeneity of the studies, random effect model was adopted and statistical heterogeneity was evaluated using the I2 test while Egger test 15 was used to detect for possible publication bias.

## Results

### Characteristics of included studies

**Figure 1:**
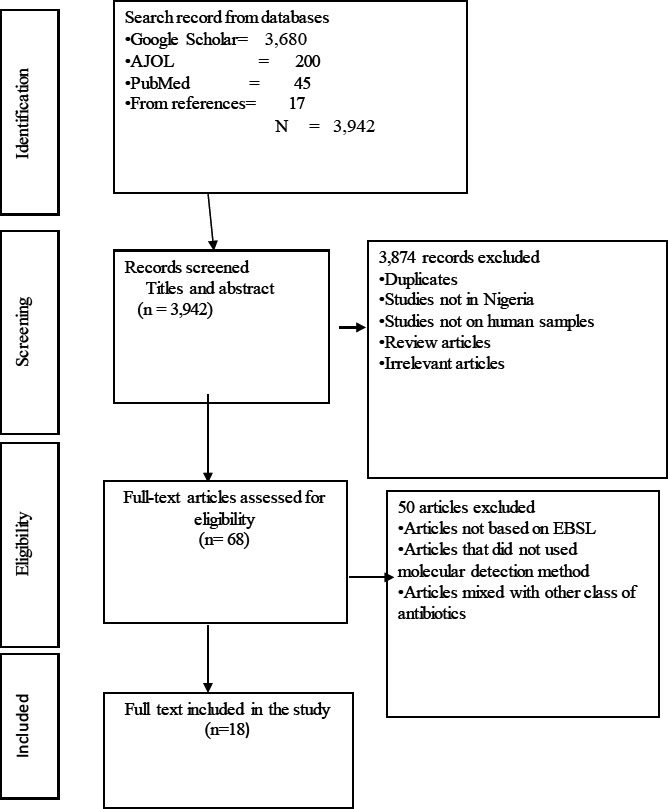
PRISMA flowchart for the selection and screening of eligible studies

A total of 3,942 studies were initially identified through the search record from electronic databases (Google scholar= 3,680, AJOL = 200, and PubMed = 45). Screened article's title and abstracts reduced eligible articles to 68 for full text evaluation. After reading the full texts, 50 articles were excluded because: their report was not based on ESBL-KP, studies did not use molecular detection method, and studies mixed with other class of antibiotics without differentiation. Thus, 18 studies met the inclusion criteria (Fig. 1).

About 3,574 *K pneumonia* isolates were screened for ESBL production, of which 1,386 ESBL were detected. Isolates source include: urine, blood, wound, and mixed clinical samples. Characteristics of included studies are summarized in Table 1.

**Table 1:** Characteristics of included studies with molecular genes detection

Author/year	Study area	Study period	Isolate source	No. of *K pneumonia* iso lates	No. of ESB L-KP	Phenotypic detection method	ESBL-KP genes			
				
							CTX-M	SHV	TEM	OXA
Ogbolu et al. 2018 16	Obomoso/Osogbo		Clinical isolates	19	11	DDST	10	8	-	9
Adeyemo et al, 2020 17	Osogbo	Jan – July	Clinical isolate	29	13	CDT	3	2	6	-
Uyanga et al, 2020 18	Akwa Ibom	July – Dec 2018	Urine	34	20	DDST	5	7	7	-
Ungo- Kore et al, 2019 19	Sokoto	3 months	Clinical isolate	13	3	DDST	3	1	2	-
Olowe et al, 2012 20	Ibadan	Oct 2010 – April 2011	Clinical isolate	66	40	DDST	9	8	8	-
Ogbolu et al, 2013 21	Ibadan	2010-2011	Fecal	60	58	DDST	20	15	58	2
Soge et al, 2006 22	Southwest	2002 – 2003	Urine	30	30	IEF	17	27	23	-
Egwuatu et al, 2019 23	Ikeja	May – July 2017	Urine	2	2	DDST	2	1	-	-
Jesumirhewe et al, 2020 24	Edo	March – May 2015	Clinical isolate	46	26	Etest	14	15	-	-
Ugbo et al, 2020 25	Abakaliki	Dec 2016 – Nov 2017	Urine	84	7	DDST	1	2	3	-
Olowo-Okere et al, 2020 26	Sokoto	Jan – July 2019	Clinical isolates	102	28	DDST	10	5	4	-
Yarima et al, 2020 27	Gombe	Sept 18 – April 2019	Urine	109	59	DDST	20 (15)	20 (15)	20 (2)	-
Mohammed et al, 2016 28	Borno	Jan – June 2014	Clinical isolates	267	80	DDST	15	10	13	-
Raji et al, 2015 29	Lagos	October 2011	Clinical isolate	30	12	Etest	10	2	9	-
Ibtihaj et al, 2021 30	Kaduna		Urine	34	4	DDST	2	2	-	-
Afolayan et al, 2021 31	Ibadan	2016 – 2018	Clinical isolate	39	28	DDST	28	-	-	-
Ugwu et al, 2020 32	Awka	June 2016 – Feb 2017	Urine	14	9	DDST	-	5	7	5
Enyinnaya et al, 2021 33	Abuja		Clinical isolate	400	114	DDST	99	78	88	-

Key: DDST- Double disk synergy test, IEF- isoelectric focusing, CDT- combination disk test

### Prevalence of ESBL-KP and genes

**Figure 2:**
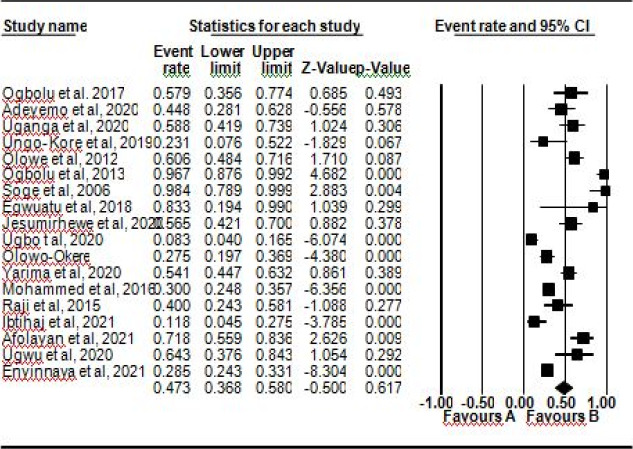
Forest plot showing pooled prevalence of ESBL-KP

**Figure 3:**
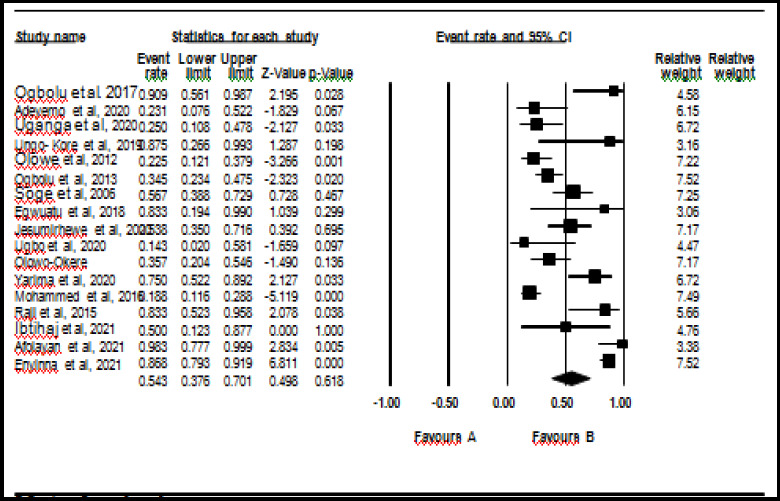
Forest plot showing pooled prevalence of ESBL-KP CTX-M genes

**Figure 4:**
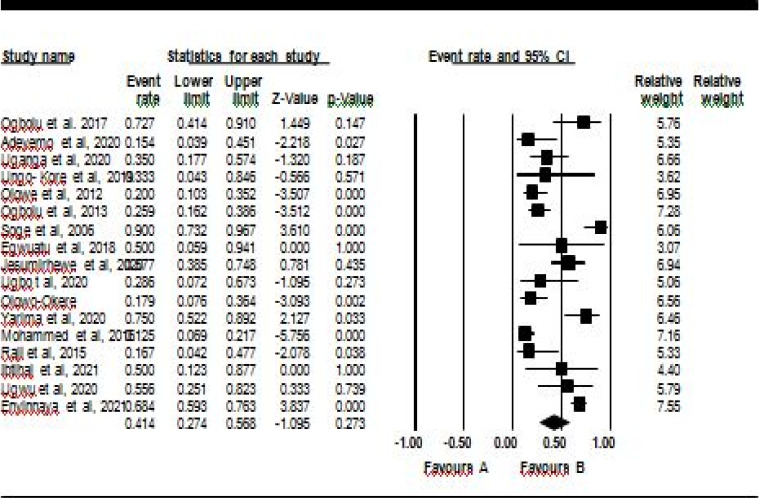
Forest plot showing pooled prevalence of ESBL-KP SHV genes

Results from the meta-analysis showed the pooled prevalence of ESBL-KP from clinical isolates was 47.3% (CI) 37 – 58%]. Among the genes encoding ESBL, pooled prevalence of CTX-M, SHV, TEM, and OXA were found to be 54% (95% CI 38, 70), 41% (95% CI 27, 57), 55% (95% CI 36, 70), and 57% (95% CI 32, 76), respectively. Forest plots showing the pooled prevalence of ESBL-KP, CTX-M and SHV genes are shown in Fig 2, 3 and 4 respectively. Heterogeneity tests (I2) was observed to be between 69.22 and 95.63 % for ESBL-KP and ESBL genes. Egger tests showed no publication bias (0.09 – 0.99) (Table 2).

**Table 2:** Meta-analysis of ESBL-KP and ESBL genes

ESBL/genes	Number of studies	Prevalence (95% CI)	a/B	I^2^ test (%) P ≤ 0.01	Egger's test
ESBL-KP	54	40 (34, 46)	3574/1374	94.10	0.094
CTX-M	17	54 (38, 70)	263/782	92.90	0.903
SHV	17	41 (27, 57)	203/763	89.45	0.989
TEM	13	55 (36, 70)	248/710	95.27	0.862
OXA	3	57 (32, 76)	38/78	69.22	0.380

Key: CI= confidence interval, a= no of ESBL-KP isolates and ESBL genes, B= total number of *K. pneumonia*

## Discussion

This review evaluated the pooled prevalence of extended-spectrum beta-lactamase-producing *Klebsiella pneumonia* and their encoding genes in Nigeria. From the literature search, this is the first systematic review and meta-analysis that is basically directed to obtain the pooled prevalence of ESBL-KP and ESBL encoding genes in Nigeria. From the results of this study, pooled prevalence of ESBL-KP in Nigeria is 47.3% (95% CI 37, ^58^). This is worrisome especially in a country like Nigeria were the health care system is not functioning perfectly and also infections due to ESBL-KP are associated with prolonged hospital stay, high cost of treatment and significantly high morbidity and mortality 34. The dissemination of ESBL-KP can occur either by direct contact from patient to patient or indirect transmission through surrounding sources and reservoirs in the environment.

Prevalence of ESBL in developed countries is lower when compared to that of the developing countries like Nigeria. The result from this study showed significantly higher prevalence of ESBL-KP than studies from Sweden in 2018, Spain (2017), and Canada (2010-2012), that reported 2%, 7.2%, and 3.6% respectively 1, 35, 36. Developing countries have reported higher prevalence. This can be illustrated by recent systematic review and meta-analysis conducted by Abrar et al. in Parkintan that reported high prevalence of 40% for ESBL producing enteriobacteriaceae in 2018 37. Also, Beigverdi et al. in 2019 conducted a meta-analysis that reported a high pooled prevalence (43.5%) of ESBL-KP in Iran. It has already been noted that the beta-lactam class of antibiotics are commonly use in the treatment of *Klebsiella* species related infections. Hence, it can be said that prior exposure to antibiotics, especially cephalosporins is an associated risk factor for the accumulation of EBSL-producing-KP^38^. In association with other factors, infections caused by ESBL producing *K. pneumonia* can result in fatal outcomes (morbidity and mortality).

The variation in prevalence of ESBL-KP between developed countries and developing countries can be attributed to the fact that policies to restrict the misuse of antibiotics are implemented in developed nations whilst inappropriate use of antibiotics is common in developing nations like Nigeria 39-41. In addition, poor microbiological laboratory facilities and usage in which the importance of detection and awareness of ESBL producing organisms are lacking contribute to the high incidence of ESBL-KP. These make physicians to prescribe drugs without adequate sensitivity tests in order to choose the most appreciate antibiotics against ESBL producing organisms^42^. Other factors that also contribute to the increased dissemination of ESBL-KP include: poor infection control, inadequate training of health workers, and poor antimicrobial stewardship 40, 41, 42-46.

In general, ESBL evolve mostly through point mutations in the beta-lactamase genes (blaSHV-1, blaTEM-1, and blaTEM-2 47. Several clinically significant ESBL enzymes such as SHV, CTX-M and TEM variants belong to the Ambler Class A; which is a classification of beta-lactamases based on amino acid sequence 48. From the results of molecular detection of ESBL-KP genes, OXA genes had the highest prevalence rate at 57%, followed by TEM (55%), CTX-M (54%), and SHV (41%); although OXA genes were detected in just 3 studies. This is because OXA is less studied among the others and it belongs to Ambler class D. This is in agreement with the systematic review on prevalence of ESBL producing enteriobacteriaceae reported by Nuhu et al. in Nigeria 11. These genes are also reported in many other countries around the world 49-52. ESBL genes are commonly carried in the plasmid which enables their dissemination. A crucial tool for bacterial survival and persistence in the face of environmental obstacles is their capacity to exchange mobile genetic element across various bacterial species and share genes between various DNA molecules 53. The prevalence of the four popular genes could be affected by the number of ESBL-KP and the number of studies used. This could be seen in the heterogeneity of the studies.

While the studies on prevalence of ESBL-KP are increasing in Nigeria, however, there are still regions where the cases are under reported. This is a serious limitation which hinders the reporting of full prevalence of ESBL-KP in Nigeria. From the characteristics of the included studies, it is obvious that the phenotypic detection is more prevalent than the molecular detection. This can be attributed to lack of resources and technical knowhow to combine phenotypic method and molecular methods by researchers. However, it is encouraging that the molecular method is fast rising due to the fact that most studies that included the molecular methods are recent studies.

## Conclusion

This systematic review and meta-analysis demonstrated that the prevalence of ESBL-KP is increasing in Nigeria. Hence, it is imperative to identify and monitor ESBL-KP strains using proper phenotypic and molecular detection methods and also antimicrobial stewardship and infection control measures for the prevention and spread of these strains should be implemented.
